# Crystal structures of two new isocoumarin derivatives: 8-amino-6-methyl-3,4-diphenyl-1*H*-isochromen-1-one and 8-amino-3,4-diethyl-6-methyl-1*H*-isochromen-1-one

**DOI:** 10.1107/S2056989019009435

**Published:** 2019-07-09

**Authors:** S. Syed Abuthahir, M. NizamMohideen, S. Mayakrishnan, N. Uma Maheswari, V. Viswanathan

**Affiliations:** a PG & Research Department of Physics, The New College (Autonomous), University of Madras, Chennai 600 014, Tamil Nadu, India; b Organic & Bioorganic Chemistry, CSIR–Central Leather Research Institute, Chennai 600 020, Tamilnadu, India; c Organic & Bioorganic Chemistry, CSIR-Central Leather Research Institute, Chennai 600 020, Tamilnadu, India; d Department of Biophysics, All India Institute of Medical Science, New Delhi 110 029, India

**Keywords:** crystal structure, chromen, isochromene, hydrogen bonding, N—H⋯π inter­actions, C—H⋯π inter­actions, offset π—π inter­actions, Hirshfeld surface analysis

## Abstract

The title compounds, 8-amino-6-methyl-3,4-diphenyl-1*H*-isochromen-1-one, C_22_H_17_NO_2_, (I), and 8-amino-3,4-diethyl-6-methyl-1*H*-isochromen-1-one, C_14_H_17_NO_2_, (II), are new isocoumarin derivatives in which the isochromene ring systems are planar. Compound II crystallizes with two independent mol­ecules (*A* and *B*) in the asymmetric unit. In I, the two phenyl rings are inclined to each other by 56.41 (7)° and to the mean plane of the 1*H*-isochromene ring system by 67.64 (6) and 44.92 (6)°. In both compounds, there is an intra­molecular N—H⋯O hydrogen bond present forming an *S*(6) ring motif. In the crystal of I, mol­ecules are linked by N—H⋯π inter­actions, forming chains along the *b*-axis direction. A C—H⋯π inter­action links the chains to form layers parallel to (100). The layers are then linked by a second C—H⋯π inter­action, forming a three-dimensional structure. In the crystal of II, the two independent mol­ecules (*A* and *B*) are linked by N—H⋯O hydrogen bonds, forming *–A–B–A–B–* chains along the [101] direction. The chains are linked into ribbons by C—H⋯π inter­actions involving inversion-related *A* mol­ecules. The latter are linked by offset π–π inter­actions [inter­centroid distances vary from 3.506 (1) to 3.870 (2) Å], forming a three-dimensional structure.

## Abstract 2

The crystal structures of two new isocoumarin derivatives, 8-amino-6-methyl-3,4-diphenyl-1*H*-isochromen-1-one and 8-amino-3,4-diethyl-6-methyl-1*H*-isochromen-1-one, are described. The inter­molecular contacts in the crystals were analysed using Hirshfeld surface analysis and two-dimensional fingerprint plots.

## Chemical context   

In recent years, there has been growing inter­est in the synthesis of natural products, since they are a tremendous and trustworthy source for the development of new drugs. The isocoumarin nucleus is a rich structural pattern in natural products (Barry, 1964 ▸) that are also constructive inter­mediates in the synthesis of a range of significant compounds, including some carbocyclic and heterocyclic compounds. Many isocoumarins show evidence of attention-grabbing biological properties and a number of pharmacological activities, such as anti­bacterial, anti­fungal, anti­tumor, anti-inflammatory, anti-allergic anti-cancer, anti-virus and anti-HIV (Khan *et al.*, 2010 ▸) activities. Isocoumarins are isolated in a enormous range of microorganisms, plants, insects and show significant biological activity, such the regulation of plant growth (Bianchi *et al.*, 2004 ▸). Isocoumarins and their derivatives are secondary metabolites of an extensive range of microbial plant and insect sources and in the creation of other medicinal compounds (Manivel *et al.*, 2008 ▸; Basvanag *et al.*, 2009 ▸). Depending on their chemical composition and concentration, they can be active either as inhibitors or stimulators in these processes. Isocoumarins and their derivatives (Ercole *et al.*, 2009 ▸; Schnebel *et al.*, 2003 ▸; Schmalle *et al.*, 1982 ▸) have been reported that have a close resemblance as far as isochromane and its attached phenyl ring is considered. The synthesis and pharmacological and other properties of coumarin and isocoumarin derivatives have been studied intensely and reviewed (Jain *et al.*, 2012 ▸; Pal *et al.*, 2011 ▸). Against this background and in view of the importance of their natural occurrence, biological activities, pharmacological activities, medicinal activities and utility as synthetic inter­mediates, we have synthesized the title compounds, and report herein on their crystal structures.
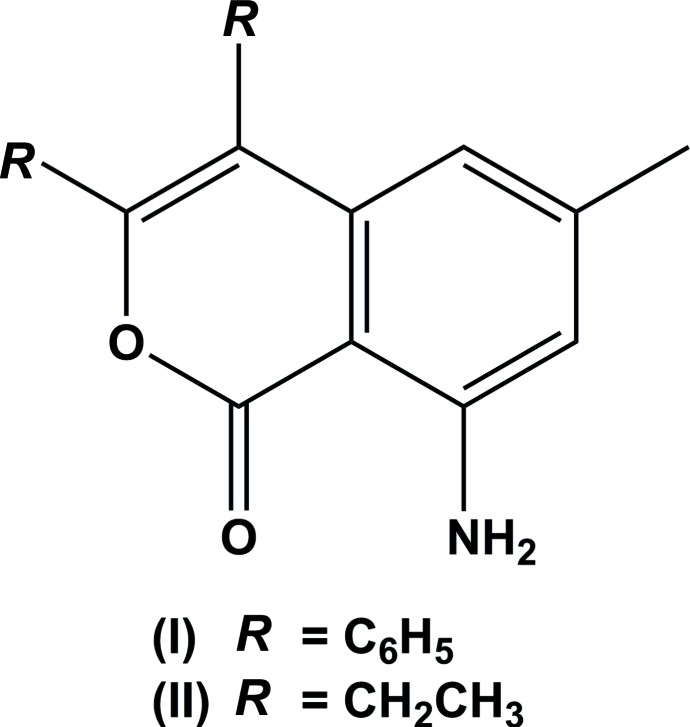


## Structural commentary   

**Figure 1:**
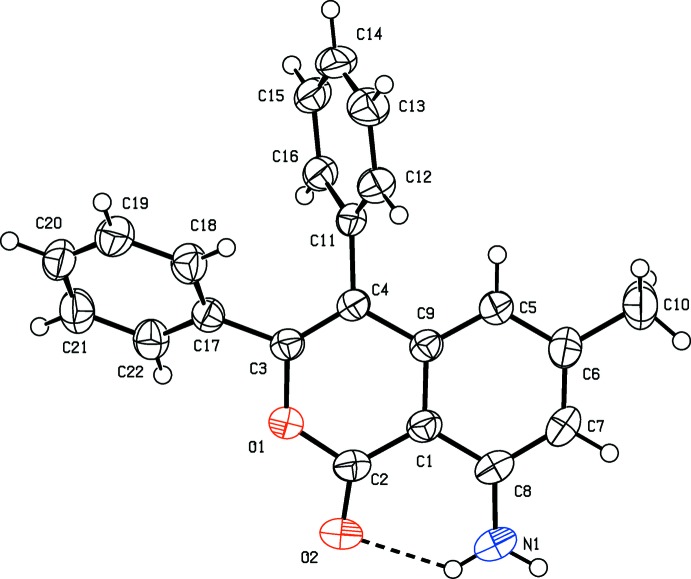
The mol­ecular structure of I, with the atom labelling. Displacement ellipsoids are drawn at the 50% probability level. The intra­molecular N—H⋯O hydrogen bond (Table 1 ▸) is shown as a dashed line.

**Figure 2:**
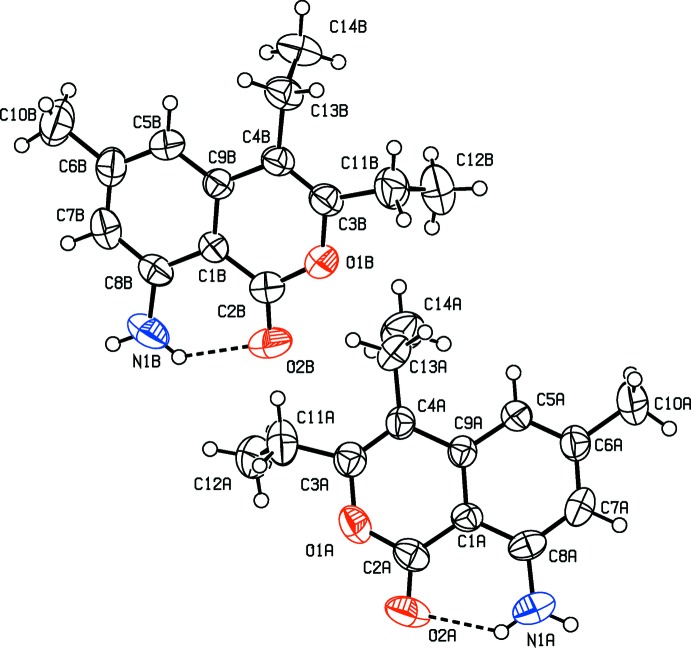
The mol­ecular structure of the two independent mol­ecules (*A* and *B*) of II, with the atom labelling. Displacement ellipsoids are drawn at the 50% probability level. The intra­molecular N—H⋯O hydrogen bonds (Table 2 ▸) are shown as dashed lines.

The mol­ecular structure and conformation of compound I is illustrated in Fig. 1 ▸. It consists of a 1*H*-isochromen-1-one moiety substituted by two phenyl groups, an amino group and a methyl group. The mol­ecular structures and conformations of the two independent mol­ecules (*A* and *B*) of compound II are illustrated in Fig. 2 ▸. Both mol­ecules consist of a 1*H*-isochromen-1-one moiety substituted by two ethyl groups, an amino group and a methyl group. The bond lengths and angles in the two independent mol­ecules agree with each other within experimental error. The normal probability plot analyses (Inter­national Tables for X-ray Crystallography, 1974, Vol. IV, pp. 293–309) for both bond lengths and angles show that the differences between the two symmetry-independent mol­ecules are of a statistical nature. For both compounds, the bond lengths and angles are close to those observed for a similar structure (Mayakrishnan *et al.*, 2018 ▸). In both compounds, there is an intra­molecular N—H⋯O hydrogen bond present in each mol­ecule forming an *S*(6) ring motif: see Table 1 ▸ and Fig. 1 ▸ for I, and Table 2 ▸ and Fig. 2 ▸ for II.

In I, the phenyl rings (C11–C16 and C17–C22) are inclined to each other by 56.41 (7)° and to the mean plane of the 1*H*-isochromen-1-one (O1/C1–C9) ring system by 67.64 (6) and 44.92 (6)°, respectively. The 1*H*-isochromen-1-one moiety is planar (r.m.s. deviation = 0.021 Å) and atom O2 deviates from the mean plane by 0.041 (1) Å. In II, the 1*H*-isochromen-1-one ring system in each mol­ecule (*A* and *B*) is also planar (r.m.s. deviations are 0.012 and 0.0321Å, respectively) and atoms O2*A* and O2*B* deviate from their respective mean planes by 0.052 (2) and 0.014 (2) Å, respectively.

## Supra­molecular features   

**Figure 3:**
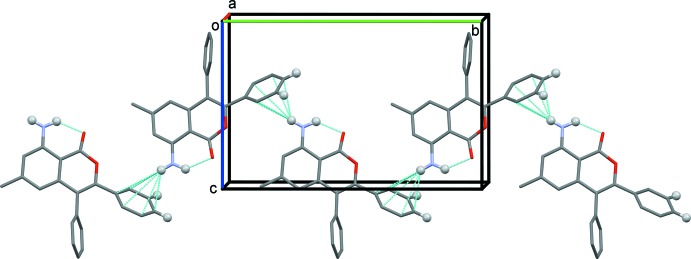
A partial view along the *a* axis of the crystal packing of I. The intra­molecular hydrogen bond and the N—H⋯π inter­action (Table 1 ▸) are shown as dashed lines, and only the H atoms (grey balls) involved in the various inter­actions have been included.

**Figure 4:**
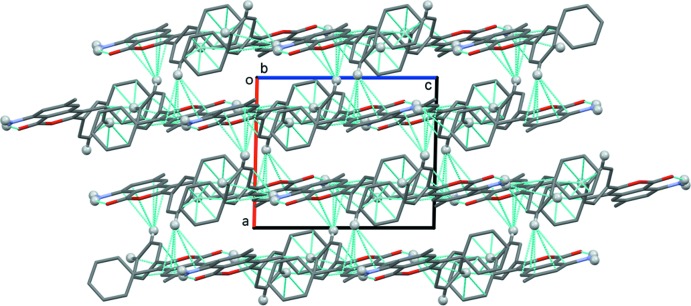
A view along the *b* axis of the crystal packing of I. The intra­molecular hydrogen bonds and the N—H⋯π and C—H⋯π inter­actions (Table 1 ▸) are shown as dashed lines, and only the H atoms (grey balls) involved in the various inter­actions have been included.

In the crystal of I, mol­ecules are linked by N—H⋯π inter­actions, forming chains along the *b*-axis direction (Fig. 3 ▸ and Table 1 ▸). A C—H⋯π inter­action (C20—H20⋯*Cg*2^ii^; Table 1 ▸) links the chains into layers parallel to (100). The layers are linked by a second C—H⋯π inter­action (C21—H21⋯*Cg*3^iii^; Table 1 ▸) to form a three-dimensional structure (Fig. 4 ▸). No significant π–π inter­actions with centroid–centroid distances less than 4 Å are observed.

**Figure 5:**
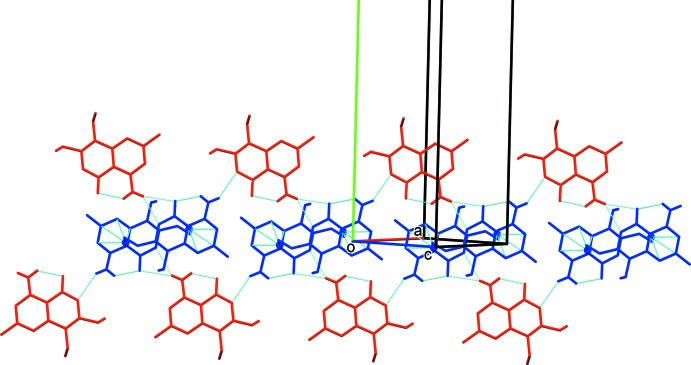
A partial view of the crystal packing of II (mol­ecule *A* blue, mol­ecule *B* red). The intra­molecular hydrogen bond (Table 2 ▸) and the C—H⋯π inter­action, involving atom H12*A* (blue ball), are shown as dashed lines, and only the H atoms involved in the various inter­actions have been included.

**Figure 6:**
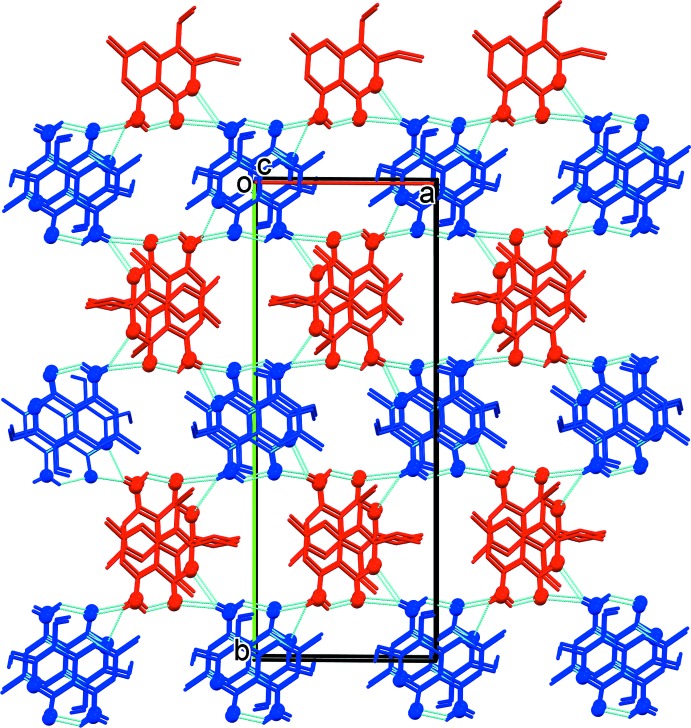
A view along the *a* axis of the crystal packing of II (mol­ecule *A* blue, mol­ecule *B* red; O and N atoms are shown as balls). The hydrogen bonds (Table 2 ▸) are shown as dashed lines, and only the H atoms involved in hydrogen bonding have been included.

In the crystal of II, the two independent mol­ecules are linked by N—H⋯O hydrogen bonds involving the amino H atom of mol­ecule *B* and the keto and chromen group oxygen atoms, O1*A* and O2*A*, of mol­ecule *A*, forming –*A*–*B*–*A*–*B*– chains along the [101] direction (see Table 2 ▸ and Fig. 5 ▸). The chains are linked by C—H⋯π inter­actions involving inversion-related *A* mol­ecules to form ribbons (Table 2 ▸ and Fig. 5 ▸). The ribbons are linked by offset π–π inter­actions, forming a three-dimensional structure (Fig. 6 ▸): inter­centroid distances *Cg*1⋯*Cg*2^i^ = 3.506 (2) Å [α = 0.97 (12)°, β = 15.9°, inter­planar distances = 3.356 (1) and 3.373 (1) Å, offset = 0.958 Å] and *Cg*3⋯*Cg*4^iv^ = 3.870 (2) Å [α = 6.01 (13)°, β = 16.5°, inter­planar distances = 3.611 (1) and 3.711 (1) Å, offset = 1.392 Å]; symmetry codes: (i) −*x*, −*y*, −*z*; (iv) −*x*, −*y* + 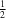, *z* − 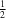; *Cg*1, *Cg*2, *Cg*3 and *Cg*4 are centroids of the (O1*A*/C1*A*–C4*A*/C9*A*), (C1*A*/C5*A*–C9*A*), (O1*B*/C1*B*–C4*B*/C9*B*) and (C1*B*/C5*B*–C9*B*) rings, respectively].

## Hirshfeld surface analysis   

The Hirshfeld surface analysis (Spackman & Jayatilaka, 2009 ▸), and the associated two-dimensional fingerprint plots (McKinnon *et al.*, 2007 ▸), to analyse the inter­molecular contacts in the crystals, were performed with *CrystalExplorer17* (Turner *et al.*, 2017 ▸).

**Figure 7:**
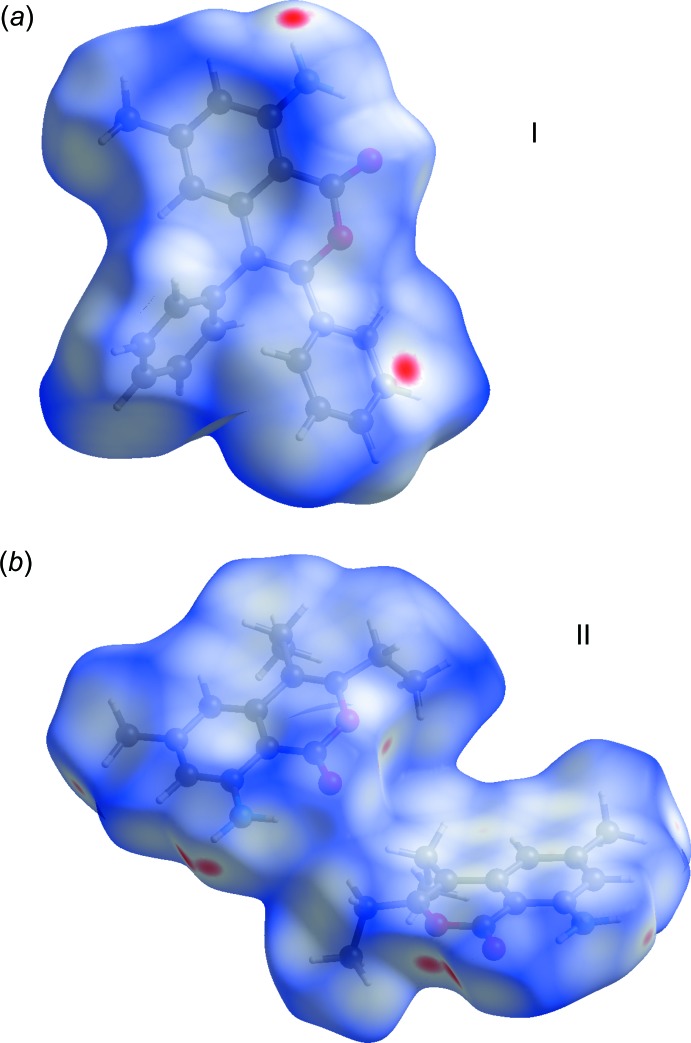
The Hirshfeld surfaces mapped over *d*_norm_, for (*a*) I and (*b*) II.

**Figure 8:**
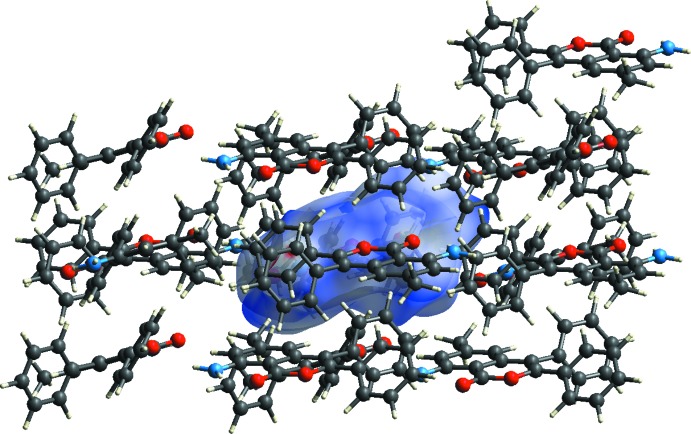
A view of the Hirshfeld surface mapped over *d*_norm_ of I, showing the various inter­molecular contacts in the crystal.

**Figure 9:**
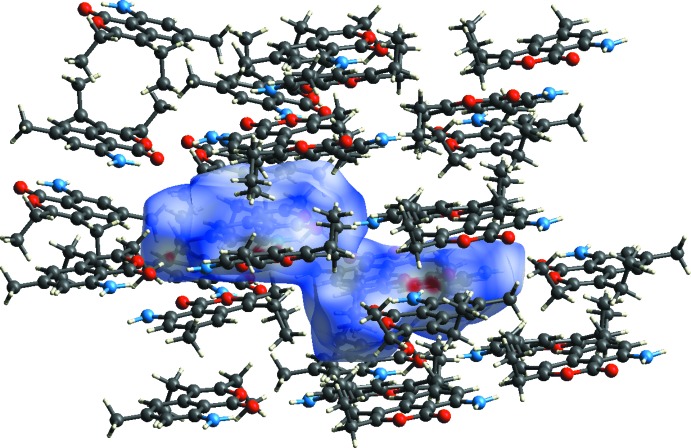
A view of the Hirshfeld surface mapped over *d*_norm_ of II, showing the various inter­molecular contacts in the crystal.

The Hirshfeld surfaces of I and II mapped over *d*_norm_ are given in Fig. 7 ▸, and the inter­molecular contacts are illustrated in Fig. 8 ▸ for I and Fig. 9 ▸ for II. They are colour-mapped with the normalized contact distance, *d*_norm_, ranging from red (distances shorter than the sum of the van der Waals radii) through white to blue (distances longer than the sum of the van der Waals radii). The *d*_norm_ surface was mapped over an arbitrary colour scale of −0.125 (red) to 1.528 (blue) for compound I and −0.178 (red) to 1.537 (blue) for compound II. The red spots on the surface indicate the inter­molecular contacts involved in hydrogen bonding.

**Figure 10:**
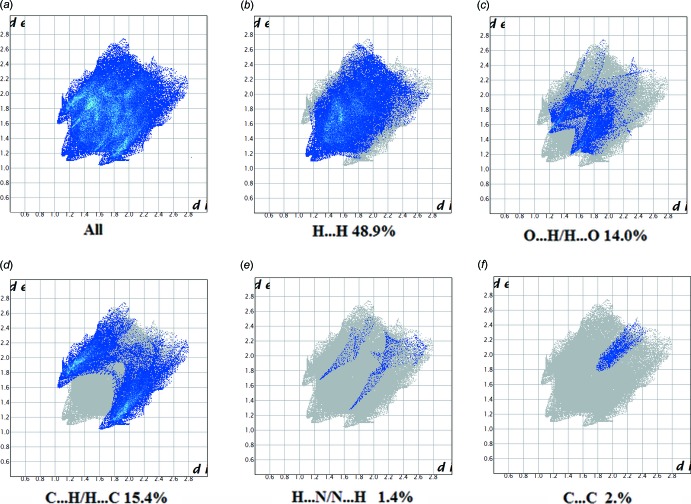
The full two-dimensional fingerprint plot for I, and fingerprint plots delineated into (*b*) H⋯H, (*c*) O⋯H/H⋯O, (*d*) C⋯H/H⋯C, (*e*) N⋯H/H⋯N contacts and (*f*) C⋯C.

**Figure 11:**
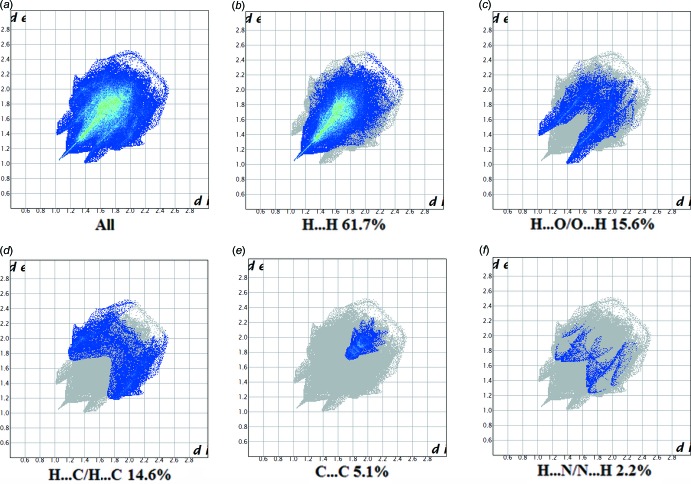
The full two-dimensional fingerprint plot for II, and fingerprint plots delineated into (*b*) H⋯H, (*c*) O⋯H/H⋯O, (*d*) C⋯·H/H⋯C, (*e*) C⋯C and (*f*) N⋯H/H⋯N contacts.

The fingerprint plots are given in Figs. 10 ▸ and 11 ▸. For I, they reveal that the principal inter­molecular contacts are H⋯H at 48.9% (Fig. 10 ▸ *b*), O⋯H/H⋯O at 14.0% (Fig. 10 ▸ *c*), C⋯H/H⋯C contacts at 15.4% (Fig. 10 ▸ *d*) and H⋯N/N⋯H at 1.4% (Fig. 10 ▸ *e*) followed by the C⋯C contacts at 2% (Fig. 10 ▸ *f*). For II, they reveal a similar trend, with the principal inter­molecular contacts being H⋯H at 61.7% (Fig. 11 ▸ *b*), O⋯H/H⋯O at 15.6% (Fig. 11 ▸ *c*), C⋯H/H⋯C contacts at 14.6% (Fig. 11 ▸ *d*), and C⋯C contacts at 5.1% (Fig. 11 ▸ *e*) followed by the H⋯N/N⋯H at 2.2% (Fig. 11 ▸ *f*). In both compounds, the H⋯H inter­molecular contacts predominate, followed by O⋯H/H⋯O contacts. However, the C⋯C contacts are significantly different: 2% *cf*. 5.1% for I and II, respectively.

## Database survey   

A search of the Cambridge Structural Database (CSD, Version 5.40, last update May 2019; Groom *et al.*, 2016 ▸) for 8-amino-1*H*-isochromen-1-ones gave only one hit, *viz*. 8-amino-3,4-bis­(4-meth­oxy­phen­yl)-1*H*-isochromen-1-one (CSD refcode NIKMAY; Mayakrishnan *et al.*, 2018 ▸). The conformation of this mol­ecule is slightly different from that of compound (I). The isochromen-1-one ring system is planar (r.m.s. deviation = 0.042 Å) and the 4-meth­oxy­phenyl rings are inclined to this mean plane by 67.22 (13) and 71.26 (11)°, and to each other by 66.91 (18)°. The corresponding dihedral angles in compound I are 67.64 (6), 44.92 (6) and 56.41 (7)°. There is an intra­molecular N—H⋯O hydrogen bond forming an *S*(6) ring motif as in compound (I). In the crystal, however, mol­ecules are linked by N—H⋯O hydrogen bonds into chains along [301], similar to the situation in compound II, rather than by N—H⋯π inter­actions as in the crystal of compound I.

## Synthesis and crystallization   

**Compound I:** An oven-dried round-bottom 25 ml flask with a magnetic stirrer bar was charged with 7-methyl-2*H*-benzo[*d*][1,3]oxazine-2,4(1*H*)-dione (1.0 equiv), di­phenyl­acetyl­ene (1.2 equiv), [RhCp*Cl_2_]_2_ (3.0 mol %), Cu(OAc) (1.0 equiv) and di­methyl­formamide (5 ml). The flask was sealed using a Teflon-coated screw cap and the reaction was continuously heated at 383 K for 24 h. The mixture was then cooled to ambient temperature, diluted with 25 ml of ethyl acetate, filtered through a celite pad, and washed with 40–60 ml of ethyl acetate. The combined organic phases were concentrated under reduced pressure, and the residue was purified by column chromatography using silica gel which led to the desired product, compound I.

**Compound II:** An oven-dried round-bottom 25 ml flask with a magnetic stirrer bar was charged with 7-methyl-2*H*-benzo[*d*][1,3]oxazine-2,4(1*H*)-dione (1.0 equiv), hex-3-yne (1.2 equiv), [RhCp*Cl_2_]_2_ (3.0 mol %), Cu(OAc) (1.0 equiv) and di­methyl­formamide (5 ml). The flask was sealed using a Teflon-coated screw cap and the reaction was continuously heated at 383 K for 24 h. The mixture was then cooled to ambient temperature, diluted with 25 ml of ethyl acetate, then filtered through a celite pad and washed with 40–60 ml of ethyl acetate. The combined organic phases were concentrated under reduced pressure, and the residue was purified by column chromatography using silica gel, which led to the desired product, *viz*. compound II.

Colourless block-like crystals of compounds I and II were obtained by slow evaporation of solutions in ethanol.

## Refinement   

Crystal data, data collection and structure refinement details are summarized in Table 3 ▸. All H atoms were positioned geometrically, with N—H = 0.86 Å, C—H = 0.93–0.97 Å, and constrained to ride on their parent atoms with *U*_iso_(H) = 1.5*U*_eq_(C-meth­yl) and 1.2*U*_eq_(N, C) for other H atoms. The crystal of compound II diffracted extremely weakly beyond 20° in θ and the data set was restricted to a maximum θ angle of 23.8°.

## Supplementary Material

Crystal structure: contains datablock(s) global, I, II. DOI: 10.1107/S2056989019009435/su5498sup1.cif

Structure factors: contains datablock(s) I. DOI: 10.1107/S2056989019009435/su5498Isup4.hkl

Structure factors: contains datablock(s) II. DOI: 10.1107/S2056989019009435/su5498IIsup5.hkl

Supporting information file. DOI: 10.1107/S2056989019009435/su5498Isup4.cml

Supporting information file. DOI: 10.1107/S2056989019009435/su5498IIsup5.cml

CCDC references: 1937678, 1937677

Additional supporting information:  crystallographic information; 3D view; checkCIF report

## Figures and Tables

**Table 1:** Hydrogen-bond geometry (Å, °) for I[Chem scheme1] *Cg*1, *Cg*2 and *Cg*3 are the centroids of the C17–C22, C11–C16 and C1/C5–C9 rings, respectively.

*D*—H⋯*A*	*D*—H	H⋯*A*	*D*⋯*A*	*D*—H⋯*A*
N1—H2*N*⋯O2	0.86	2.05	2.6915 (19)	131
N1—H1*N*⋯*Cg*1^i^	0.86	2.81	3.631 (2)	157
C20—H20⋯*Cg*2^ii^	0.93	2.70	3.588 (2)	160
C21—H21⋯*Cg*3^iii^	0.93	2.84	3.488 (2)	128

Symmetry codes: (i) 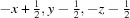; (ii) 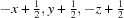; (iii) 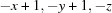.

**Table 2:** Hydrogen-bond geometry (Å, °) for II[Chem scheme1] *Cg*2 is the centroid of the C1*A*/C5*A*–C9*A* ring.

*D*—H⋯*A*	*D*—H	H⋯*A*	*D*⋯*A*	*D*—H⋯*A*
N1*A*—H1*A*2⋯O2*A*	0.86	2.05	2.701 (3)	131
N1*B*—H1*B*2⋯O2*B*	0.86	2.05	2.696 (3)	131
N1*A*—H1*A*1⋯O1*B* ^i^	0.86	2.57	3.328 (3)	148
N1*B*—H1*B*1⋯O1*A* ^ii^	0.86	2.50	3.235 (3)	143
N1*B*—H1*B*1⋯O2*A* ^ii^	0.86	2.53	3.367 (3)	165
C12*A*—H12*A*⋯*Cg*2^iii^	0.96	2.99	3.773 (2)	140

Symmetry codes: (i) 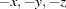; (ii) 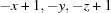; (iii) 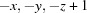.

**Table 3:** Experimental details

	I	II
Crystal data		
Chemical formula	C_22_H_17_NO_2_	C_14_H_17_NO_2_
*M*_r_	327.36	231.28
Crystal system, space group	Monoclinic, *P*2_1_/*n*	Monoclinic, *P*2_1_/*c*
Temperature (K)	296	296
*a*, *b*, *c* (Å)	9.1652 (3), 16.9764 (6), 10.9687 (4)	10.4844 (8), 26.562 (2), 9.3651 (6)
β (°)	91.156 (1)	105.367 (3)
*V* (Å^3^)	1706.30 (10)	2514.8 (3)
*Z*	4	8
Radiation type	Mo *K*α	Mo *K*α
μ (mm^−1^)	0.08	0.08
Crystal size (mm)	0.32 × 0.18 × 0.12	0.25 × 0.22 × 0.13

Data collection		
Diffractometer	Bruker Kappa APEXII CCD	Bruker Kappa APEXII CCD
Absorption correction	Multi-scan (*SADABS*; Bruker, 2008 ▸)	Multi-scan (*SADABS*; Bruker, 2008 ▸)
*T*_min_, *T*_max_	0.756, 0.824	0.756, 0.824
No. of measured, independent and observed [*I* > 2σ(*I*)] reflections	14904, 3628, 2719	13067, 3755, 2132
*R*_int_	0.025	0.048
θ_max_ (°)	26.8	23.8
(sin θ/λ)_max_ (Å^−1^)	0.634	0.567

Refinement		
*R*[*F* ^2^ > 2σ(*F* ^2^)], *wR*(*F* ^2^), *S*	0.040, 0.118, 1.03	0.054, 0.168, 1.01
No. of reflections	3628	3755
No. of parameters	228	313
H-atom treatment	H-atom parameters constrained	H-atom parameters constrained
Δρ_max_, Δρ_min_ (e Å^−3^)	0.22, −0.15	0.15, −0.25

Computer programs: *APEX2* and *SAINT* (Bruker, 2008 ▸), *SHELXS2018* (Sheldrick, 2008 ▸), *SHELXL2018* (Sheldrick, 2015 ▸), *ORTEP-3 for Windows* and *WinGX* (Farrugia, 2012 ▸), *Mercury* (Macrae *et al.*, 2008 ▸), *publCIF* (Westrip, 2010 ▸) and *PLATON* (Spek, 2009 ▸).

## supplementary crystallographic information

### 8-Amino-6-methyl-3,4-diphenyl-1H-isochromen-1-one (I) Crystal data

**Table:**

C_22_H_17_NO_2_	*F*(000) = 688
*M**_r_* = 327.36	*D*_x_ = 1.274 Mg m^−^^3^
Monoclinic, *P*2_1_/*n*	Mo *K*α radiation, λ = 0.71073 Å
*a* = 9.1652 (3) Å	Cell parameters from 3001 reflections
*b* = 16.9764 (6) Å	θ = 1.8–26.9°
*c* = 10.9687 (4) Å	µ = 0.08 mm^−^^1^
β = 91.156 (1)°	*T* = 296 K
*V* = 1706.30 (10) Å^3^	Block, colourless
*Z* = 4	0.32 × 0.18 × 0.12 mm

### 8-Amino-6-methyl-3,4-diphenyl-1H-isochromen-1-one (I) Data collection

**Table:**

Bruker Kappa APEXII CCD diffractometer	2719 reflections with *I* > 2σ(*I*)
ω and φ scans	*R*_int_ = 0.025
Absorption correction: multi-scan (*SADABS*; Bruker, 2008)	θ_max_ = 26.8°, θ_min_ = 2.2°
*T*_min_ = 0.756, *T*_max_ = 0.824	*h* = −7→11
14904 measured reflections	*k* = −16→21
3628 independent reflections	*l* = −13→13

### 8-Amino-6-methyl-3,4-diphenyl-1H-isochromen-1-one (I) Refinement

**Table:**

Refinement on *F*^2^	Secondary atom site location: difference Fourier map
Least-squares matrix: full	Hydrogen site location: inferred from neighbouring sites
*R*[*F*^2^ > 2σ(*F*^2^)] = 0.040	H-atom parameters constrained
*wR*(*F*^2^) = 0.118	*w* = 1/[σ^2^(*F*_o_^2^) + (0.0561*P*)^2^ + 0.3498*P*] where *P* = (*F*_o_^2^ + 2*F*_c_^2^)/3
*S* = 1.03	(Δ/σ)_max_ = 0.001
3628 reflections	Δρ_max_ = 0.22 e Å^−^^3^
228 parameters	Δρ_min_ = −0.15 e Å^−^^3^
0 restraints	Extinction correction: (SHELXL-2018/3; Sheldrick 2015), Fc^*^=kFc[1+0.001xFc^2^λ^3^/sin(2θ)]^-1/4^
Primary atom site location: structure-invariant direct methods	Extinction coefficient: 0.027 (3)

### 8-Amino-6-methyl-3,4-diphenyl-1H-isochromen-1-one (I) Special details

**Table:**

Geometry. All esds (except the esd in the dihedral angle between two l.s. planes) are estimated using the full covariance matrix. The cell esds are taken into account individually in the estimation of esds in distances, angles and torsion angles; correlations between esds in cell parameters are only used when they are defined by crystal symmetry. An approximate (isotropic) treatment of cell esds is used for estimating esds involving l.s. planes.

### 8-Amino-6-methyl-3,4-diphenyl-1H-isochromen-1-one (I) Fractional atomic coordinates and isotropic or equivalent isotropic displacement parameters (Å^2^)

**Table:**

	*x*	*y*	*z*	*U*_iso_*/*U*_eq_	
O1	0.30517 (11)	0.50819 (6)	−0.13908 (8)	0.0428 (3)	
O2	0.34168 (15)	0.45740 (7)	−0.31876 (9)	0.0642 (4)	
N1	0.29626 (19)	0.30274 (9)	−0.35783 (12)	0.0693 (5)	
H1N	0.296082	0.260175	−0.400359	0.083*	
H2N	0.321068	0.346662	−0.390514	0.083*	
C1	0.25918 (14)	0.36948 (8)	−0.16525 (11)	0.0369 (3)	
C2	0.30400 (16)	0.44376 (9)	−0.21526 (12)	0.0423 (3)	
C3	0.26388 (14)	0.50381 (8)	−0.01861 (11)	0.0343 (3)	
C4	0.22498 (14)	0.43536 (8)	0.03282 (11)	0.0328 (3)	
C5	0.18557 (15)	0.29115 (8)	0.00781 (13)	0.0404 (3)	
H5	0.162684	0.287498	0.089835	0.048*	
C6	0.18321 (16)	0.22339 (9)	−0.06446 (14)	0.0444 (4)	
C7	0.21712 (16)	0.22931 (9)	−0.18611 (14)	0.0466 (4)	
H7	0.213012	0.184295	−0.234375	0.056*	
C8	0.25737 (16)	0.30058 (9)	−0.23912 (12)	0.0437 (4)	
C9	0.22152 (14)	0.36370 (8)	−0.04094 (11)	0.0338 (3)	
C10	0.1460 (2)	0.14520 (10)	−0.00914 (18)	0.0717 (6)	
H10A	0.063444	0.151158	0.042499	0.108*	
H10B	0.227983	0.126275	0.038250	0.108*	
H10C	0.122961	0.108174	−0.072811	0.108*	
C11	0.19198 (14)	0.43298 (7)	0.16566 (11)	0.0341 (3)	
C12	0.05157 (16)	0.41920 (9)	0.20535 (13)	0.0427 (3)	
H12	−0.022264	0.406927	0.149128	0.051*	
C13	0.02070 (18)	0.42361 (10)	0.32806 (14)	0.0523 (4)	
H13	−0.074106	0.415271	0.353831	0.063*	
C14	0.1301 (2)	0.44033 (10)	0.41240 (13)	0.0530 (4)	
H14	0.108969	0.443448	0.494830	0.064*	
C15	0.27035 (18)	0.45239 (9)	0.37447 (13)	0.0471 (4)	
H15	0.344296	0.463112	0.431370	0.057*	
C16	0.30160 (16)	0.44859 (8)	0.25200 (12)	0.0395 (3)	
H16	0.396814	0.456537	0.226936	0.047*	
C17	0.27264 (14)	0.58255 (8)	0.03882 (12)	0.0359 (3)	
C18	0.16250 (17)	0.60991 (9)	0.11333 (14)	0.0465 (4)	
H18	0.081520	0.578437	0.127440	0.056*	
C19	0.1727 (2)	0.68360 (9)	0.16655 (15)	0.0556 (4)	
H19	0.098591	0.701582	0.216101	0.067*	
C20	0.2924 (2)	0.73034 (9)	0.14627 (14)	0.0547 (4)	
H20	0.299569	0.779622	0.182915	0.066*	
C21	0.40126 (18)	0.70442 (9)	0.07209 (15)	0.0527 (4)	
H21	0.482036	0.736185	0.058695	0.063*	
C22	0.39120 (16)	0.63116 (9)	0.01716 (14)	0.0447 (4)	
H22	0.464196	0.614417	−0.034512	0.054*	

### 8-Amino-6-methyl-3,4-diphenyl-1H-isochromen-1-one (I) Atomic displacement parameters (Å^2^)

**Table:**

	*U*^11^	*U*^22^	*U*^33^	*U*^12^	*U*^13^	*U*^23^
O1	0.0583 (6)	0.0377 (6)	0.0324 (5)	−0.0033 (5)	0.0039 (4)	0.0015 (4)
O2	0.1014 (10)	0.0597 (8)	0.0317 (6)	−0.0104 (7)	0.0110 (6)	0.0030 (5)
N1	0.1126 (13)	0.0608 (9)	0.0347 (7)	−0.0103 (9)	0.0097 (7)	−0.0132 (6)
C1	0.0402 (7)	0.0394 (8)	0.0310 (6)	0.0003 (6)	−0.0027 (5)	−0.0023 (5)
C2	0.0523 (8)	0.0445 (8)	0.0301 (7)	0.0001 (7)	−0.0013 (6)	−0.0002 (6)
C3	0.0358 (7)	0.0364 (7)	0.0307 (6)	0.0014 (6)	−0.0008 (5)	−0.0007 (5)
C4	0.0340 (6)	0.0339 (7)	0.0305 (6)	0.0023 (5)	−0.0008 (5)	−0.0023 (5)
C5	0.0477 (8)	0.0367 (8)	0.0370 (7)	−0.0016 (6)	0.0064 (6)	−0.0021 (6)
C6	0.0440 (8)	0.0362 (8)	0.0531 (8)	−0.0033 (6)	0.0050 (6)	−0.0064 (6)
C7	0.0508 (8)	0.0414 (9)	0.0474 (8)	−0.0016 (7)	−0.0002 (7)	−0.0158 (7)
C8	0.0486 (8)	0.0495 (9)	0.0329 (7)	−0.0003 (7)	−0.0020 (6)	−0.0088 (6)
C9	0.0347 (7)	0.0351 (7)	0.0316 (6)	0.0009 (6)	−0.0017 (5)	−0.0019 (5)
C10	0.0957 (14)	0.0402 (10)	0.0803 (13)	−0.0122 (9)	0.0301 (11)	−0.0094 (9)
C11	0.0424 (7)	0.0271 (7)	0.0328 (7)	0.0027 (6)	0.0010 (5)	−0.0023 (5)
C12	0.0422 (7)	0.0455 (9)	0.0404 (8)	0.0012 (6)	0.0027 (6)	−0.0027 (6)
C13	0.0547 (9)	0.0536 (10)	0.0493 (9)	0.0054 (8)	0.0173 (7)	0.0003 (7)
C14	0.0782 (11)	0.0486 (9)	0.0327 (7)	0.0133 (8)	0.0101 (7)	−0.0031 (6)
C15	0.0650 (10)	0.0406 (8)	0.0354 (7)	0.0070 (7)	−0.0083 (7)	−0.0049 (6)
C16	0.0444 (7)	0.0373 (8)	0.0367 (7)	0.0019 (6)	−0.0020 (6)	−0.0015 (6)
C17	0.0401 (7)	0.0320 (7)	0.0355 (7)	0.0032 (6)	−0.0066 (6)	0.0020 (5)
C18	0.0486 (8)	0.0389 (8)	0.0520 (9)	0.0026 (7)	0.0036 (7)	−0.0011 (7)
C19	0.0721 (11)	0.0435 (9)	0.0513 (9)	0.0128 (9)	0.0072 (8)	−0.0047 (7)
C20	0.0816 (12)	0.0332 (8)	0.0488 (9)	0.0013 (8)	−0.0110 (8)	−0.0042 (7)
C21	0.0570 (9)	0.0399 (9)	0.0609 (10)	−0.0069 (7)	−0.0110 (8)	0.0020 (7)
C22	0.0440 (8)	0.0394 (8)	0.0505 (8)	0.0015 (7)	−0.0020 (6)	0.0003 (6)

### 8-Amino-6-methyl-3,4-diphenyl-1H-isochromen-1-one (I) Geometric parameters (Å, º)

**Table:**

O1—C2	1.3763 (17)	C11—C12	1.3867 (19)
O1—C3	1.3838 (15)	C11—C16	1.3922 (19)
O2—C2	1.2155 (16)	C12—C13	1.383 (2)
N1—C8	1.3574 (19)	C12—H12	0.9300
N1—H1N	0.8600	C13—C14	1.380 (2)
N1—H2N	0.8600	C13—H13	0.9300
C1—C9	1.4167 (18)	C14—C15	1.374 (2)
C1—C8	1.4227 (19)	C14—H14	0.9300
C1—C2	1.438 (2)	C15—C16	1.3807 (19)
C3—C4	1.3430 (18)	C15—H15	0.9300
C3—C17	1.4792 (18)	C16—H16	0.9300
C4—C9	1.4611 (18)	C17—C22	1.389 (2)
C4—C11	1.4947 (17)	C17—C18	1.391 (2)
C5—C9	1.3851 (19)	C18—C19	1.383 (2)
C5—C6	1.397 (2)	C18—H18	0.9300
C5—H5	0.9300	C19—C20	1.375 (2)
C6—C7	1.380 (2)	C19—H19	0.9300
C6—C10	1.501 (2)	C20—C21	1.373 (2)
C7—C8	1.395 (2)	C20—H20	0.9300
C7—H7	0.9300	C21—C22	1.384 (2)
C10—H10A	0.9600	C21—H21	0.9300
C10—H10B	0.9600	C22—H22	0.9300
C10—H10C	0.9600		
			
C2—O1—C3	122.56 (11)	C12—C11—C16	118.69 (12)
C8—N1—H1N	120.0	C12—C11—C4	121.20 (12)
C8—N1—H2N	120.0	C16—C11—C4	120.04 (12)
H1N—N1—H2N	120.0	C13—C12—C11	120.39 (14)
C9—C1—C8	119.40 (13)	C13—C12—H12	119.8
C9—C1—C2	120.31 (12)	C11—C12—H12	119.8
C8—C1—C2	120.25 (12)	C14—C13—C12	120.22 (15)
O2—C2—O1	114.63 (13)	C14—C13—H13	119.9
O2—C2—C1	127.72 (13)	C12—C13—H13	119.9
O1—C2—C1	117.64 (11)	C15—C14—C13	119.94 (14)
C4—C3—O1	121.84 (12)	C15—C14—H14	120.0
C4—C3—C17	128.00 (12)	C13—C14—H14	120.0
O1—C3—C17	110.16 (11)	C14—C15—C16	120.08 (14)
C3—C4—C9	119.40 (11)	C14—C15—H15	120.0
C3—C4—C11	119.57 (11)	C16—C15—H15	120.0
C9—C4—C11	120.98 (11)	C15—C16—C11	120.64 (14)
C9—C5—C6	120.94 (13)	C15—C16—H16	119.7
C9—C5—H5	119.5	C11—C16—H16	119.7
C6—C5—H5	119.5	C22—C17—C18	118.79 (13)
C7—C6—C5	119.18 (14)	C22—C17—C3	120.00 (12)
C7—C6—C10	120.84 (14)	C18—C17—C3	121.20 (13)
C5—C6—C10	119.98 (14)	C19—C18—C17	120.41 (15)
C6—C7—C8	122.20 (13)	C19—C18—H18	119.8
C6—C7—H7	118.9	C17—C18—H18	119.8
C8—C7—H7	118.9	C20—C19—C18	120.06 (15)
N1—C8—C7	119.99 (14)	C20—C19—H19	120.0
N1—C8—C1	121.59 (14)	C18—C19—H19	120.0
C7—C8—C1	118.41 (13)	C21—C20—C19	120.17 (15)
C5—C9—C1	119.84 (12)	C21—C20—H20	119.9
C5—C9—C4	121.96 (12)	C19—C20—H20	119.9
C1—C9—C4	118.19 (12)	C20—C21—C22	120.21 (15)
C6—C10—H10A	109.5	C20—C21—H21	119.9
C6—C10—H10B	109.5	C22—C21—H21	119.9
H10A—C10—H10B	109.5	C21—C22—C17	120.32 (14)
C6—C10—H10C	109.5	C21—C22—H22	119.8
H10A—C10—H10C	109.5	C17—C22—H22	119.8
H10B—C10—H10C	109.5		
			
C3—O1—C2—O2	179.19 (13)	C3—C4—C9—C5	178.47 (13)
C3—O1—C2—C1	−0.67 (19)	C11—C4—C9—C5	1.05 (19)
C9—C1—C2—O2	178.61 (15)	C3—C4—C9—C1	−0.26 (18)
C8—C1—C2—O2	0.9 (2)	C11—C4—C9—C1	−177.68 (12)
C9—C1—C2—O1	−1.5 (2)	C3—C4—C11—C12	111.95 (15)
C8—C1—C2—O1	−179.26 (12)	C9—C4—C11—C12	−70.64 (17)
C2—O1—C3—C4	2.49 (19)	C3—C4—C11—C16	−64.84 (17)
C2—O1—C3—C17	−178.45 (12)	C9—C4—C11—C16	112.57 (14)
O1—C3—C4—C9	−1.94 (19)	C16—C11—C12—C13	2.2 (2)
C17—C3—C4—C9	179.17 (12)	C4—C11—C12—C13	−174.66 (13)
O1—C3—C4—C11	175.51 (11)	C11—C12—C13—C14	−1.2 (2)
C17—C3—C4—C11	−3.4 (2)	C12—C13—C14—C15	−0.2 (2)
C9—C5—C6—C7	−0.1 (2)	C13—C14—C15—C16	0.7 (2)
C9—C5—C6—C10	179.11 (15)	C14—C15—C16—C11	0.3 (2)
C5—C6—C7—C8	1.6 (2)	C12—C11—C16—C15	−1.7 (2)
C10—C6—C7—C8	−177.57 (16)	C4—C11—C16—C15	175.14 (13)
C6—C7—C8—N1	176.95 (15)	C4—C3—C17—C22	136.31 (15)
C6—C7—C8—C1	−1.8 (2)	O1—C3—C17—C22	−42.68 (16)
C9—C1—C8—N1	−178.25 (14)	C4—C3—C17—C18	−44.8 (2)
C2—C1—C8—N1	−0.5 (2)	O1—C3—C17—C18	136.26 (13)
C9—C1—C8—C7	0.5 (2)	C22—C17—C18—C19	−1.2 (2)
C2—C1—C8—C7	178.22 (13)	C3—C17—C18—C19	179.82 (13)
C6—C5—C9—C1	−1.2 (2)	C17—C18—C19—C20	−0.2 (2)
C6—C5—C9—C4	−179.90 (13)	C18—C19—C20—C21	0.7 (2)
C8—C1—C9—C5	0.97 (19)	C19—C20—C21—C22	0.1 (2)
C2—C1—C9—C5	−176.77 (12)	C20—C21—C22—C17	−1.5 (2)
C8—C1—C9—C4	179.73 (12)	C18—C17—C22—C21	2.0 (2)
C2—C1—C9—C4	1.99 (19)	C3—C17—C22—C21	−178.99 (13)

### 8-Amino-6-methyl-3,4-diphenyl-1H-isochromen-1-one (I) Hydrogen-bond geometry (Å, º)

*Cg*1, *Cg*2 and *Cg*3 are the centroids of the C17–C22, C11–C16 and C1/C5–C9 rings, respectively.

**Table:**

*D*—H···*A*	*D*—H	H···*A*	*D*···*A*	*D*—H···*A*
N1—H2*N*···O2	0.86	2.05	2.6915 (19)	131
N1—H1*N*···*Cg*1^i^	0.86	2.81	3.631 (2)	157
C20—H20···*Cg*2^ii^	0.93	2.70	3.588 (2)	160
C21—H21···*Cg*3^iii^	0.93	2.84	3.488 (2)	128

Symmetry codes: (i) −*x*+1/2, *y*−1/2, −*z*+3/2; (ii) −*x*+1/2, *y*+1/2, −*z*+1/2; (iii) −*x*, −*y*+2, −*z*+1.

### 8-Amino-3,4-diethyl-6-methyl-1H-isochromen-1-one (II) Crystal data

**Table:**

C_14_H_17_NO_2_	*F*(000) = 992
*M**_r_* = 231.28	*D*_x_ = 1.222 Mg m^−^^3^
Monoclinic, *P*2_1_/*c*	Mo *K*α radiation, λ = 0.71073 Å
*a* = 10.4844 (8) Å	Cell parameters from 3755 reflections
*b* = 26.562 (2) Å	θ = 1.8–26.9°
*c* = 9.3651 (6) Å	µ = 0.08 mm^−^^1^
β = 105.367 (3)°	*T* = 296 K
*V* = 2514.8 (3) Å^3^	Block, colourless
*Z* = 8	0.25 × 0.22 × 0.13 mm

### 8-Amino-3,4-diethyl-6-methyl-1H-isochromen-1-one (II) Data collection

**Table:**

Bruker Kappa APEXII CCD diffractometer	2132 reflections with *I* > 2σ(*I*)
ω and φ scans	*R*_int_ = 0.048
Absorption correction: multi-scan (*SADABS*; Bruker, 2008)	θ_max_ = 23.8°, θ_min_ = 2.2°
*T*_min_ = 0.756, *T*_max_ = 0.824	*h* = −11→11
13067 measured reflections	*k* = −29→23
3755 independent reflections	*l* = −10→7

### 8-Amino-3,4-diethyl-6-methyl-1H-isochromen-1-one (II) Refinement

**Table:**

Refinement on *F*^2^	Primary atom site location: structure-invariant direct methods
Least-squares matrix: full	Secondary atom site location: difference Fourier map
*R*[*F*^2^ > 2σ(*F*^2^)] = 0.054	Hydrogen site location: inferred from neighbouring sites
*wR*(*F*^2^) = 0.168	H-atom parameters constrained
*S* = 1.01	*w* = 1/[σ^2^(*F*_o_^2^) + (0.0872*P*)^2^] where *P* = (*F*_o_^2^ + 2*F*_c_^2^)/3
3755 reflections	(Δ/σ)_max_ < 0.001
313 parameters	Δρ_max_ = 0.14 e Å^−^^3^
0 restraints	Δρ_min_ = −0.25 e Å^−^^3^

### 8-Amino-3,4-diethyl-6-methyl-1H-isochromen-1-one (II) Special details

**Table:**

Geometry. All esds (except the esd in the dihedral angle between two l.s. planes) are estimated using the full covariance matrix. The cell esds are taken into account individually in the estimation of esds in distances, angles and torsion angles; correlations between esds in cell parameters are only used when they are defined by crystal symmetry. An approximate (isotropic) treatment of cell esds is used for estimating esds involving l.s. planes.

### 8-Amino-3,4-diethyl-6-methyl-1H-isochromen-1-one (II) Fractional atomic coordinates and isotropic or equivalent isotropic displacement parameters (Å^2^)

**Table:**

	*x*	*y*	*z*	*U*_iso_*/*U*_eq_	
O1A	0.19526 (18)	−0.03883 (8)	0.3169 (2)	0.0621 (6)	
O2A	0.1114 (2)	−0.10719 (9)	0.2007 (2)	0.0765 (7)	
N1A	−0.1310 (2)	−0.10445 (9)	0.0078 (2)	0.0688 (7)	
H1A1	−0.198031	−0.117045	−0.055946	0.083*	
H1A2	−0.062249	−0.122669	0.044001	0.083*	
C1A	−0.0241 (2)	−0.03391 (10)	0.1564 (2)	0.0420 (7)	
C2A	0.0926 (3)	−0.06286 (12)	0.2204 (3)	0.0529 (8)	
C3A	0.1906 (3)	0.01162 (12)	0.3555 (3)	0.0532 (8)	
C4A	0.0834 (2)	0.03978 (10)	0.3010 (3)	0.0458 (7)	
C5A	−0.1438 (3)	0.04469 (10)	0.1329 (3)	0.0493 (7)	
H5A	−0.147825	0.078312	0.159015	0.059*	
C6A	−0.2512 (3)	0.02321 (12)	0.0321 (3)	0.0538 (8)	
C7A	−0.2452 (3)	−0.02619 (13)	−0.0072 (3)	0.0556 (8)	
H7A	−0.317482	−0.040423	−0.074901	0.067*	
C8A	−0.1338 (3)	−0.05578 (11)	0.0513 (3)	0.0492 (7)	
C9A	−0.0303 (2)	0.01716 (10)	0.1956 (2)	0.0417 (7)	
C10A	−0.3711 (3)	0.05465 (13)	−0.0348 (3)	0.0806 (10)	
H10A	−0.349955	0.079114	−0.100375	0.121*	
H10B	−0.398847	0.071563	0.042527	0.121*	
H10C	−0.441292	0.033370	−0.089177	0.121*	
C11A	0.3183 (3)	0.02507 (13)	0.4640 (3)	0.0761 (10)	
H11A	0.326355	0.061432	0.470382	0.091*	
H11B	0.391030	0.012236	0.428775	0.091*	
C12A	0.3281 (3)	0.00403 (13)	0.6162 (3)	0.0828 (11)	
H12A	0.262132	0.019401	0.656040	0.124*	
H12B	0.414351	0.010997	0.679928	0.124*	
H12C	0.314202	−0.031714	0.609490	0.124*	
C13A	0.0781 (3)	0.09389 (11)	0.3488 (3)	0.0597 (8)	
H13A	0.033743	0.113977	0.263609	0.072*	
H13B	0.167579	0.106592	0.385238	0.072*	
C14A	0.0062 (3)	0.09996 (13)	0.4687 (3)	0.0818 (10)	
H14A	−0.082689	0.087702	0.433030	0.123*	
H14B	0.004438	0.134906	0.494236	0.123*	
H14C	0.051468	0.081108	0.554672	0.123*	
O1B	0.34202 (18)	0.19483 (8)	0.14816 (19)	0.0623 (6)	
O2B	0.4302 (2)	0.12582 (9)	0.2580 (2)	0.0859 (7)	
N1B	0.6446 (2)	0.13134 (10)	0.4921 (3)	0.0786 (8)	
H1B1	0.705569	0.119291	0.564264	0.094*	
H1B2	0.591805	0.111350	0.431977	0.094*	
C1B	0.5316 (2)	0.20312 (11)	0.3560 (3)	0.0442 (7)	
C2B	0.4371 (3)	0.17168 (12)	0.2564 (3)	0.0562 (8)	
C3B	0.3370 (3)	0.24666 (12)	0.1273 (3)	0.0532 (8)	
C4B	0.4230 (3)	0.27743 (10)	0.2151 (3)	0.0479 (7)	
C5B	0.6132 (3)	0.28607 (11)	0.4388 (3)	0.0564 (8)	
H5B	0.608867	0.320854	0.427094	0.068*	
C6B	0.7082 (3)	0.26519 (14)	0.5561 (3)	0.0614 (8)	
C7B	0.7174 (3)	0.21419 (14)	0.5708 (3)	0.0628 (9)	
H7B	0.782810	0.200493	0.648214	0.075*	
C8B	0.6315 (3)	0.18187 (11)	0.4730 (3)	0.0531 (8)	
C9B	0.5246 (2)	0.25581 (11)	0.3387 (3)	0.0447 (7)	
C10B	0.8005 (3)	0.29914 (14)	0.6652 (4)	0.0914 (12)	
H10D	0.861250	0.279047	0.737701	0.137*	
H10E	0.750228	0.320132	0.713663	0.137*	
H10F	0.848950	0.319861	0.613864	0.137*	
C11B	0.2212 (3)	0.25846 (13)	0.0000 (3)	0.0767 (10)	
H11C	0.226498	0.238303	−0.084589	0.092*	
H11D	0.225174	0.293610	−0.026570	0.092*	
C12B	0.0907 (3)	0.24846 (15)	0.0340 (4)	0.1083 (14)	
H12D	0.090713	0.214998	0.072749	0.162*	
H12E	0.020240	0.251665	−0.055014	0.162*	
H12F	0.078047	0.272358	0.105949	0.162*	
C13B	0.4150 (3)	0.33380 (11)	0.1925 (3)	0.0604 (8)	
H13C	0.374007	0.340940	0.088909	0.073*	
H13D	0.503755	0.347615	0.217150	0.073*	
C14B	0.3362 (3)	0.35947 (11)	0.2865 (3)	0.0731 (9)	
H14D	0.247412	0.346648	0.260456	0.110*	
H14E	0.334475	0.395109	0.269095	0.110*	
H14F	0.376919	0.352867	0.389239	0.110*	

### 8-Amino-3,4-diethyl-6-methyl-1H-isochromen-1-one (II) Atomic displacement parameters (Å^2^)

**Table:**

	*U*^11^	*U*^22^	*U*^33^	*U*^12^	*U*^13^	*U*^23^
O1A	0.0559 (13)	0.0674 (16)	0.0564 (12)	0.0158 (11)	0.0035 (10)	0.0115 (11)
O2A	0.1035 (18)	0.0453 (15)	0.0803 (14)	0.0221 (13)	0.0241 (12)	0.0094 (12)
N1A	0.0866 (19)	0.0476 (18)	0.0742 (17)	−0.0169 (14)	0.0249 (14)	−0.0101 (13)
C1A	0.0511 (17)	0.0357 (18)	0.0386 (13)	−0.0001 (13)	0.0105 (12)	0.0057 (12)
C2A	0.064 (2)	0.049 (2)	0.0463 (16)	0.0100 (17)	0.0163 (15)	0.0123 (15)
C3A	0.0533 (19)	0.056 (2)	0.0485 (16)	−0.0012 (16)	0.0103 (14)	0.0049 (14)
C4A	0.0430 (16)	0.049 (2)	0.0427 (14)	−0.0015 (14)	0.0061 (13)	0.0024 (13)
C5A	0.0508 (17)	0.0438 (19)	0.0486 (15)	0.0030 (14)	0.0049 (14)	0.0003 (13)
C6A	0.0471 (18)	0.059 (2)	0.0502 (16)	0.0017 (16)	0.0046 (14)	0.0033 (15)
C7A	0.0494 (18)	0.067 (2)	0.0456 (16)	−0.0171 (17)	0.0040 (13)	−0.0008 (15)
C8A	0.067 (2)	0.0393 (19)	0.0470 (15)	−0.0117 (16)	0.0243 (15)	−0.0002 (14)
C9A	0.0415 (16)	0.0449 (19)	0.0371 (13)	−0.0023 (13)	0.0078 (12)	0.0049 (12)
C10A	0.055 (2)	0.092 (3)	0.081 (2)	0.0079 (19)	−0.0065 (16)	0.0077 (19)
C11A	0.0516 (19)	0.096 (3)	0.070 (2)	−0.0024 (18)	−0.0020 (16)	0.0106 (19)
C12A	0.087 (2)	0.090 (3)	0.0563 (19)	0.007 (2)	−0.0078 (17)	−0.0083 (18)
C13A	0.0571 (18)	0.059 (2)	0.0587 (17)	−0.0130 (15)	0.0078 (14)	−0.0117 (15)
C14A	0.093 (2)	0.083 (3)	0.073 (2)	−0.002 (2)	0.0271 (18)	−0.0218 (18)
O1B	0.0632 (13)	0.0536 (15)	0.0614 (12)	−0.0025 (11)	0.0012 (10)	−0.0087 (11)
O2B	0.1026 (18)	0.0414 (16)	0.1027 (17)	−0.0062 (13)	0.0079 (14)	−0.0060 (13)
N1B	0.0833 (19)	0.056 (2)	0.093 (2)	0.0262 (15)	0.0168 (15)	0.0176 (15)
C1B	0.0418 (16)	0.044 (2)	0.0457 (14)	0.0047 (14)	0.0097 (13)	0.0033 (13)
C2B	0.0595 (19)	0.046 (2)	0.0612 (18)	−0.0001 (17)	0.0132 (16)	−0.0027 (16)
C3B	0.0580 (19)	0.050 (2)	0.0506 (16)	0.0081 (16)	0.0120 (14)	0.0028 (15)
C4B	0.0540 (17)	0.0415 (19)	0.0477 (15)	0.0049 (14)	0.0125 (14)	0.0031 (14)
C5B	0.0607 (19)	0.043 (2)	0.0632 (17)	−0.0024 (15)	0.0131 (16)	−0.0019 (15)
C6B	0.0448 (18)	0.074 (3)	0.0608 (18)	−0.0025 (17)	0.0062 (15)	−0.0052 (18)
C7B	0.0483 (18)	0.081 (3)	0.0538 (17)	0.0108 (18)	0.0049 (14)	0.0059 (18)
C8B	0.0516 (18)	0.048 (2)	0.0620 (18)	0.0109 (15)	0.0197 (15)	0.0048 (15)
C9B	0.0437 (16)	0.043 (2)	0.0479 (15)	0.0016 (13)	0.0124 (13)	0.0002 (13)
C10B	0.066 (2)	0.109 (3)	0.087 (2)	−0.010 (2)	−0.0018 (19)	−0.024 (2)
C11B	0.075 (2)	0.086 (3)	0.0574 (18)	0.0129 (19)	−0.0039 (17)	−0.0030 (17)
C12B	0.063 (2)	0.118 (4)	0.125 (3)	0.013 (2)	−0.009 (2)	0.008 (3)
C13B	0.073 (2)	0.052 (2)	0.0570 (16)	0.0107 (16)	0.0178 (15)	0.0086 (15)
C14B	0.092 (2)	0.050 (2)	0.083 (2)	0.0184 (18)	0.0329 (19)	0.0023 (17)

### 8-Amino-3,4-diethyl-6-methyl-1H-isochromen-1-one (II) Geometric parameters (Å, º)

**Table:**

O1A—C2A	1.366 (3)	O1B—C2B	1.365 (3)
O1A—C3A	1.392 (3)	O1B—C3B	1.389 (3)
O2A—C2A	1.216 (3)	O2B—C2B	1.221 (3)
N1A—C8A	1.358 (3)	N1B—C8B	1.356 (3)
N1A—H1A1	0.8600	N1B—H1B1	0.8600
N1A—H1A2	0.8600	N1B—H1B2	0.8600
C1A—C9A	1.411 (3)	C1B—C9B	1.409 (3)
C1A—C8A	1.425 (3)	C1B—C8B	1.417 (3)
C1A—C2A	1.435 (4)	C1B—C2B	1.435 (4)
C3A—C4A	1.334 (3)	C3B—C4B	1.328 (4)
C3A—C11A	1.494 (4)	C3B—C11B	1.492 (4)
C4A—C9A	1.461 (3)	C4B—C9B	1.467 (3)
C4A—C13A	1.511 (4)	C4B—C13B	1.511 (4)
C5A—C6A	1.386 (3)	C5B—C6B	1.388 (4)
C5A—C9A	1.387 (3)	C5B—C9B	1.388 (3)
C5A—H5A	0.9300	C5B—H5B	0.9300
C6A—C7A	1.369 (4)	C6B—C7B	1.363 (4)
C6A—C10A	1.501 (4)	C6B—C10B	1.507 (4)
C7A—C8A	1.393 (4)	C7B—C8B	1.396 (4)
C7A—H7A	0.9300	C7B—H7B	0.9300
C10A—H10A	0.9600	C10B—H10D	0.9600
C10A—H10B	0.9600	C10B—H10E	0.9600
C10A—H10C	0.9600	C10B—H10F	0.9600
C11A—C12A	1.509 (4)	C11B—C12B	1.509 (4)
C11A—H11A	0.9700	C11B—H11C	0.9700
C11A—H11B	0.9700	C11B—H11D	0.9700
C12A—H12A	0.9600	C12B—H12D	0.9600
C12A—H12B	0.9600	C12B—H12E	0.9600
C12A—H12C	0.9600	C12B—H12F	0.9600
C13A—C14A	1.517 (4)	C13B—C14B	1.519 (4)
C13A—H13A	0.9700	C13B—H13C	0.9700
C13A—H13B	0.9700	C13B—H13D	0.9700
C14A—H14A	0.9600	C14B—H14D	0.9600
C14A—H14B	0.9600	C14B—H14E	0.9600
C14A—H14C	0.9600	C14B—H14F	0.9600
			
C2A—O1A—C3A	123.1 (2)	C2B—O1B—C3B	122.9 (2)
C8A—N1A—H1A1	120.0	C8B—N1B—H1B1	120.0
C8A—N1A—H1A2	120.0	C8B—N1B—H1B2	120.0
H1A1—N1A—H1A2	120.0	H1B1—N1B—H1B2	120.0
C9A—C1A—C8A	119.1 (2)	C9B—C1B—C8B	119.3 (2)
C9A—C1A—C2A	120.0 (2)	C9B—C1B—C2B	119.8 (2)
C8A—C1A—C2A	120.9 (3)	C8B—C1B—C2B	120.8 (3)
O2A—C2A—O1A	115.0 (3)	O2B—C2B—O1B	115.1 (3)
O2A—C2A—C1A	127.6 (3)	O2B—C2B—C1B	127.3 (3)
O1A—C2A—C1A	117.5 (3)	O1B—C2B—C1B	117.6 (3)
C4A—C3A—O1A	121.6 (2)	C4B—C3B—O1B	121.9 (2)
C4A—C3A—C11A	129.7 (3)	C4B—C3B—C11B	129.8 (3)
O1A—C3A—C11A	108.7 (3)	O1B—C3B—C11B	108.3 (3)
C3A—C4A—C9A	118.8 (3)	C3B—C4B—C9B	118.6 (3)
C3A—C4A—C13A	120.9 (2)	C3B—C4B—C13B	121.3 (2)
C9A—C4A—C13A	120.3 (2)	C9B—C4B—C13B	120.0 (2)
C6A—C5A—C9A	121.5 (3)	C6B—C5B—C9B	121.0 (3)
C6A—C5A—H5A	119.3	C6B—C5B—H5B	119.5
C9A—C5A—H5A	119.3	C9B—C5B—H5B	119.5
C7A—C6A—C5A	119.4 (3)	C7B—C6B—C5B	119.6 (3)
C7A—C6A—C10A	121.0 (3)	C7B—C6B—C10B	120.7 (3)
C5A—C6A—C10A	119.7 (3)	C5B—C6B—C10B	119.7 (3)
C6A—C7A—C8A	121.9 (3)	C6B—C7B—C8B	121.9 (3)
C6A—C7A—H7A	119.0	C6B—C7B—H7B	119.0
C8A—C7A—H7A	119.0	C8B—C7B—H7B	119.0
N1A—C8A—C7A	120.1 (3)	N1B—C8B—C7B	119.8 (3)
N1A—C8A—C1A	121.2 (3)	N1B—C8B—C1B	121.6 (3)
C7A—C8A—C1A	118.7 (3)	C7B—C8B—C1B	118.6 (3)
C5A—C9A—C1A	119.3 (2)	C5B—C9B—C1B	119.5 (2)
C5A—C9A—C4A	121.6 (3)	C5B—C9B—C4B	121.5 (3)
C1A—C9A—C4A	119.1 (2)	C1B—C9B—C4B	119.0 (2)
C6A—C10A—H10A	109.5	C6B—C10B—H10D	109.5
C6A—C10A—H10B	109.5	C6B—C10B—H10E	109.5
H10A—C10A—H10B	109.5	H10D—C10B—H10E	109.5
C6A—C10A—H10C	109.5	C6B—C10B—H10F	109.5
H10A—C10A—H10C	109.5	H10D—C10B—H10F	109.5
H10B—C10A—H10C	109.5	H10E—C10B—H10F	109.5
C3A—C11A—C12A	112.3 (3)	C3B—C11B—C12B	112.7 (3)
C3A—C11A—H11A	109.1	C3B—C11B—H11C	109.1
C12A—C11A—H11A	109.1	C12B—C11B—H11C	109.1
C3A—C11A—H11B	109.1	C3B—C11B—H11D	109.1
C12A—C11A—H11B	109.1	C12B—C11B—H11D	109.1
H11A—C11A—H11B	107.9	H11C—C11B—H11D	107.8
C11A—C12A—H12A	109.5	C11B—C12B—H12D	109.5
C11A—C12A—H12B	109.5	C11B—C12B—H12E	109.5
H12A—C12A—H12B	109.5	H12D—C12B—H12E	109.5
C11A—C12A—H12C	109.5	C11B—C12B—H12F	109.5
H12A—C12A—H12C	109.5	H12D—C12B—H12F	109.5
H12B—C12A—H12C	109.5	H12E—C12B—H12F	109.5
C4A—C13A—C14A	112.6 (2)	C4B—C13B—C14B	112.5 (2)
C4A—C13A—H13A	109.1	C4B—C13B—H13C	109.1
C14A—C13A—H13A	109.1	C14B—C13B—H13C	109.1
C4A—C13A—H13B	109.1	C4B—C13B—H13D	109.1
C14A—C13A—H13B	109.1	C14B—C13B—H13D	109.1
H13A—C13A—H13B	107.8	H13C—C13B—H13D	107.8
C13A—C14A—H14A	109.5	C13B—C14B—H14D	109.5
C13A—C14A—H14B	109.5	C13B—C14B—H14E	109.5
H14A—C14A—H14B	109.5	H14D—C14B—H14E	109.5
C13A—C14A—H14C	109.5	C13B—C14B—H14F	109.5
H14A—C14A—H14C	109.5	H14D—C14B—H14F	109.5
H14B—C14A—H14C	109.5	H14E—C14B—H14F	109.5
			
C3A—O1A—C2A—O2A	−178.6 (2)	C3B—O1B—C2B—O2B	−178.2 (2)
C3A—O1A—C2A—C1A	0.4 (3)	C3B—O1B—C2B—C1B	2.8 (4)
C9A—C1A—C2A—O2A	177.9 (3)	C9B—C1B—C2B—O2B	−179.6 (3)
C8A—C1A—C2A—O2A	−3.1 (4)	C8B—C1B—C2B—O2B	−0.2 (4)
C9A—C1A—C2A—O1A	−0.9 (3)	C9B—C1B—C2B—O1B	−0.7 (4)
C8A—C1A—C2A—O1A	178.1 (2)	C8B—C1B—C2B—O1B	178.7 (2)
C2A—O1A—C3A—C4A	0.8 (4)	C2B—O1B—C3B—C4B	−2.0 (4)
C2A—O1A—C3A—C11A	179.3 (2)	C2B—O1B—C3B—C11B	179.9 (2)
O1A—C3A—C4A—C9A	−1.5 (4)	O1B—C3B—C4B—C9B	−1.0 (4)
C11A—C3A—C4A—C9A	−179.6 (2)	C11B—C3B—C4B—C9B	176.7 (3)
O1A—C3A—C4A—C13A	177.5 (2)	O1B—C3B—C4B—C13B	−179.1 (2)
C11A—C3A—C4A—C13A	−0.6 (4)	C11B—C3B—C4B—C13B	−1.4 (4)
C9A—C5A—C6A—C7A	−0.4 (4)	C9B—C5B—C6B—C7B	2.1 (4)
C9A—C5A—C6A—C10A	−179.1 (2)	C9B—C5B—C6B—C10B	−178.3 (3)
C5A—C6A—C7A—C8A	0.1 (4)	C5B—C6B—C7B—C8B	−1.7 (4)
C10A—C6A—C7A—C8A	178.7 (3)	C10B—C6B—C7B—C8B	178.8 (3)
C6A—C7A—C8A—N1A	−179.0 (2)	C6B—C7B—C8B—N1B	−180.0 (3)
C6A—C7A—C8A—C1A	0.8 (4)	C6B—C7B—C8B—C1B	−0.5 (4)
C9A—C1A—C8A—N1A	178.5 (2)	C9B—C1B—C8B—N1B	−178.3 (2)
C2A—C1A—C8A—N1A	−0.5 (4)	C2B—C1B—C8B—N1B	2.3 (4)
C9A—C1A—C8A—C7A	−1.3 (3)	C9B—C1B—C8B—C7B	2.2 (4)
C2A—C1A—C8A—C7A	179.7 (2)	C2B—C1B—C8B—C7B	−177.2 (2)
C6A—C5A—C9A—C1A	−0.2 (4)	C6B—C5B—C9B—C1B	−0.3 (4)
C6A—C5A—C9A—C4A	179.6 (2)	C6B—C5B—C9B—C4B	179.4 (2)
C8A—C1A—C9A—C5A	1.0 (3)	C8B—C1B—C9B—C5B	−1.8 (3)
C2A—C1A—C9A—C5A	−180.0 (2)	C2B—C1B—C9B—C5B	177.6 (2)
C8A—C1A—C9A—C4A	−178.7 (2)	C8B—C1B—C9B—C4B	178.5 (2)
C2A—C1A—C9A—C4A	0.2 (3)	C2B—C1B—C9B—C4B	−2.2 (3)
C3A—C4A—C9A—C5A	−178.8 (2)	C3B—C4B—C9B—C5B	−176.7 (2)
C13A—C4A—C9A—C5A	2.2 (4)	C13B—C4B—C9B—C5B	1.4 (4)
C3A—C4A—C9A—C1A	1.0 (3)	C3B—C4B—C9B—C1B	3.0 (3)
C13A—C4A—C9A—C1A	−178.1 (2)	C13B—C4B—C9B—C1B	−178.9 (2)
C4A—C3A—C11A—C12A	103.6 (4)	C4B—C3B—C11B—C12B	−109.5 (4)
O1A—C3A—C11A—C12A	−74.7 (3)	O1B—C3B—C11B—C12B	68.5 (3)
C3A—C4A—C13A—C14A	−98.1 (3)	C3B—C4B—C13B—C14B	93.8 (3)
C9A—C4A—C13A—C14A	80.9 (3)	C9B—C4B—C13B—C14B	−84.3 (3)

### 8-Amino-3,4-diethyl-6-methyl-1H-isochromen-1-one (II) Hydrogen-bond geometry (Å, º)

*Cg*1 is the centroid of the C1*A*/C5*A*–C9*A* ring.

**Table:**

*D*—H···*A*	*D*—H	H···*A*	*D*···*A*	*D*—H···*A*
N1*A*—H1*A*2···O2*A*	0.86	2.05	2.701 (3)	131
N1*B*—H1*B*2···O2*B*	0.86	2.05	2.696 (3)	131
N1*A*—H1*A*1···O1*B*^i^	0.86	2.57	3.328 (3)	148
N1*B*—H1*B*1···O1*A*^ii^	0.86	2.50	3.235 (3)	143
N1*B*—H1*B*1···O2*A*^ii^	0.86	2.53	3.367 (3)	165
C12*A*—H12*A*···*Cg*1^iii^	0.96	2.99	3.773 (2)	140

Symmetry codes: (i) −*x*, −*y*, −*z*; (ii) −*x*+1, −*y*, −*z*+1; (iii) −*x*+2, −*y*+1, −*z*.
